# Supplementary material for: Towards a unified generic framework to define and observe contacts between livestock and wildlife: a systematic review
Source: PeerJ. 2020 Oct 26;8:e10221. doi: 10.7717/peerj.10221 (PMC7594637; doi:10.7717/peerj.10221)
Supplement: Supplemental Information 1 [file peerj-08-10221-s001.docx]

**Supplementary Information 1:** Rationale and contribution of ‘Towards a unified generic framework to define and observe contacts between livestock and wildlife: A systematic review’

1. Rationale for conducting the systematic review

Disease transmission events between livestock and wildlife have the potential for major implications to human health, the economy and conservation. Transmission is dependent on direct or indirect contact between livestock and wildlife, but these contacts are difficult to observe due to the cryptic nature of many wildlife species. Knowledge of the types and frequency of livestock-wildlife contacts could improve the modelling of diseases that have multiple host species, and identify risk factors in order to predict where they are more likely to occur and prevent them. There are a range of ways that livestock-wildlife contact data can be gathered and used, and ambiguity in the definition of a contact, which means it is difficult to compare these types of studies. This systematic review was conducted to identify the reasons for collecting livestock-wildlife contact data, the observational methods used to do so, and to identify commonalities to standardise the collection and reporting of livestock-contact data.

1. The contribution that the systematic review makes to knowledge

This systematic review summarises observational methods that have been used to monitor specific types of livestock-wildlife contacts. We have considered these methods in the context of the study objectives and identified commonalities in order to propose a unified generic framework that can be used to categorise livestock-wildlife contacts that can be used for a variety of species for non-vector borne diseases. The framework will help standardise the collection and reporting of contact data, and is a step towards being able to effectively evaluate wildlife-livestock observation methods. Consistent contact reporting will be useful in the development of disease transmission models based on empirical data to more effectively mitigate disease, and will assist in study design and choice of observation method.
