# Supplementary material for: Towards a unified generic framework to define and observe contacts between livestock and wildlife: a systematic review
Source: PeerJ. 2020 Oct 26;8:e10221. doi: 10.7717/peerj.10221 (PMC7594637; doi:10.7717/peerj.10221)
Supplement: Supplemental Information 2 [file peerj-08-10221-s002.doc]

| **Section/topic** | **#** | **Checklist item** | **Reported on page #** |
| --- | --- | --- | --- |
| **TITLE** | | |  |
| Title | 1 | Identify the report as a systematic review, meta-analysis, or both. | Page 1: Towards a unified generic framework to define and observe livestock-wildlife interactions: A systematic review |
| **ABSTRACT** | | |  |
| Structured summary | 2 | Provide a structured summary including, as applicable: background; objectives; data sources; study eligibility criteria, participants, and interventions; study appraisal and synthesis methods; results; limitations; conclusions and implications of key findings; systematic review registration number. | Page 2 |
| **INTRODUCTION** | | |  |
| Rationale | 3 | Describe the rationale for the review in the context of what is already known. | Page 5: Livestock-wildlife contact data is needed to inform the simulation and modelling of diseases that have multiple host species, but information on the types of contact needed for transmission and the rates at which these occur is lacking. In the context of disease transmission, defining a contact is challenging and while types of contact are often broadly grouped into being ‘direct’ or ‘indirect’, there are no standardised definitions. This variation in definitions means it is difficult to make meaningful comparisons between studies and to apply findings from one study to different contexts. Therefore, a standardised generic template for defining livestock-wildlife contacts would be useful. |
| Objectives | 4 | Provide an explicit statement of questions being addressed with reference to participants, interventions, comparisons, outcomes, and study design (PICOS). | Page 6: The aim of this study was to systematically review the reasons for, and observational methods used in, studies investigating livestock-wildlife contacts, and to propose a generalised framework for defining contacts between livestock and wildlife.  Page 7: The systematic review question was “Which methods have been used to assess the frequency of, types of, and risk factors for, contacts between wild animals and livestock or livestock farms worldwide?”. |
| **METHODS** | | |  |
| Protocol and registration | 5 | Indicate if a review protocol exists, if and where it can be accessed (e.g., Web address), and, if available, provide registration information including registration number. | Review protocol detailed on pages 7 to 8. Not registered with an external body because not a health-related review |
| Eligibility criteria | 6 | Specify study characteristics (e.g., PICOS, length of follow-up) and report characteristics (e.g., years considered, language, publication status) used as criteria for eligibility, giving rationale. | Pages 6-7: We wished to capture publications that collected, used or analysed data to investigate direct or indirect contacts between farmed livestock and wild mammals whose adult bodyweight is typically >5kg (thus excluding studies on small rodents). Specifically, publications were included if they attempted to quantify, characterise, or identify risk factors for livestock-wildlife contacts. Only articles in English and those accessible to researchers were included. All reasonable efforts were made to access papers that passed abstract screening. We excluded studies in which predation events were the sole indicator of wildlife-livestock interactions, and studies of wild animals that were not free-living, were tamed or were relocated for the purpose of monitoring interactions. Publications until 11 November 2019 were included, and no time restrictions were applied to the start of the search. Working definitions of direct and indirect contact were agreed by the researchers, before performing the literature search, to avoid ambiguity when evaluating publications for inclusion. Working definitions were deliberately broad to capture as many potentially relevant publications as possible. Space is included as a resource that can be shared between wildlife and livestock. Time was not specifically mentioned. It can be assumed that if an animal is in physical contact with another, then it is at the same time. For the shared use of resources, no time limit was set.  Also see Table 1 (Page 30) |
| Information sources | 7 | Describe all information sources (e.g., databases with dates of coverage, contact with study authors to identify additional studies) in the search and date last searched. | Page 7: Searches were conducted in CAB Abstracts, Scopus and Pubmed on 11^th^ November 2019. |
| Search | 8 | Present full electronic search strategy for at least one database, including any limits used, such that it could be repeated. | Table S1 |
| Study selection | 9 | State the process for selecting studies (i.e., screening, eligibility, included in systematic review, and, if applicable, included in the meta-analysis). | Page 7: Titles, abstracts and full texts of the retrieved publications were evaluated by the lead author against pre-specified exclusion and inclusion criteria.  Also see Figure 1 and Table 1 |
| Data collection process | 10 | Describe method of data extraction from reports (e.g., piloted forms, independently, in duplicate) and any processes for obtaining and confirming data from investigators. | Page 8: Livestock and wildlife species, observation methods and definitions were categorised by the lead author. Studies were grouped into seven themes that emerged during data extraction and were agreed upon by the authors |
| Data items | 11 | List and define all variables for which data were sought (e.g., PICOS, funding sources) and any assumptions and simplifications made. | Page 8: Livestock and wildlife species, observation methods and definitions |
| Risk of bias in individual studies | 12 | Describe methods used for assessing risk of bias of individual studies (including specification of whether this was done at the study or outcome level), and how this information is to be used in any data synthesis. | Page 8: The quality of the selected papers was appraised against a set of criteria based on the study objectives, definitions of contact, certainty of results and robustness of conclusions (Table 1 and Figure S2). Each criterion contributed equally to the overall quality score for each paper. |
| Summary measures | 13 | State the principal summary measures (e.g., risk ratio, difference in means). | Page 8: Descriptive. Livestock and wildlife species, observation methods and definitions. Studies were grouped into seven themes that emerged during data extraction and were agreed upon by the authors |
| Synthesis of results | 14 | Describe the methods of handling data and combining results of studies, if done, including measures of consistency (e.g., I^2^) for each meta-analysis. | Pages 7-8: Excel and R |

Page 1 of 2

| **Section/topic** | **#** | **Checklist item** | **Reported on page #** |
| --- | --- | --- | --- |
| Risk of bias across studies | 15 | Specify any assessment of risk of bias that may affect the cumulative evidence (e.g., publication bias, selective reporting within studies). | Page 15: Definitions describing contacts between livestock and wildlife were found to be wide-ranging. Making any sort of meaningful comparison between such studies is challenging.  Page 16: narrow scope of livestock-wildlife contact studies, with the majority of studies focusing on cattle-wildlife contacts and diseases of cattle. Bovine tuberculosis (infection with *M. bovis*) featured prominently, indicative of the economic and potentially zoonotic importance of this disease to the USA and UK, where the most livestock-wildlife contact studies were conducted |
| Additional analyses | 16 | Describe methods of additional analyses (e.g., sensitivity or subgroup analyses, meta-regression), if done, indicating which were pre-specified. | Not done |
| **RESULTS** | | |  |
| Study selection | 17 | Give numbers of studies screened, assessed for eligibility, and included in the review, with reasons for exclusions at each stage, ideally with a flow diagram. | Page 10: A total of 43,032 papers were identified by the search terms across all three databases, of which 30,080 were unique results. After screening using the exclusion and inclusion criteria in Table 1, 122 publications remained in the final analysis  Also see Figure 1 |
| Study characteristics | 18 | For each study, present characteristics for which data were extracted (e.g., study size, PICOS, follow-up period) and provide the citations. | Page 33 (Table 4) |
| Risk of bias within studies | 19 | Present data on risk of bias of each study and, if available, any outcome level assessment (see item 12). | Figure S2 |
| Results of individual studies | 20 | For all outcomes considered (benefits or harms), present, for each study: (a) simple summary data for each intervention group (b) effect estimates and confidence intervals, ideally with a forest plot. | Not applicable in general, although specific findings reported as examples throughout the Results section (pages 10 to 13) |
| Synthesis of results | 21 | Present results of each meta-analysis done, including confidence intervals and measures of consistency. | Pages 10-14: Descriptive. Methods that monitored both livestock and wildlife species were used in 88 publications (72%) whereas 34 studies (28%) monitored wildlife only. Camera trapping was the most used method of monitoring wildlife (37 studies, 31%), and was most prominently used in badgers, deer and wild pigs (Figure 3). GPS collars were the second most used method to monitor wildlife (29 studies, 24%), and while they were also used predominantly on badgers, deer and wild pigs, they were used proportionally more than cameras to monitor predators and large herbivores such as buffalo, wild horses and elephants. Other methods used to monitor wildlife were direct observation (21; 17%), farmer questionnaires (20; 16%), radio-transmitters (17; 14%), activity signs (15; 12%) and proximity loggers (7; 6%). |
| Risk of bias across studies | 22 | Present results of any assessment of risk of bias across studies (see Item 15). | Page 15: Definitions describing contacts between livestock and wildlife were found to be wide-ranging. Making any sort of meaningful comparison between such studies is challenging.  Page 16: narrow scope of livestock-wildlife contact studies, with the majority of studies focusing on cattle-wildlife contacts and diseases of cattle. Bovine tuberculosis (infection with *M. bovis*) featured prominently, indicative of the economic and potentially zoonotic importance of this disease to the USA and UK, where the most livestock-wildlife contact studies were conducted |
| Additional analysis | 23 | Give results of additional analyses, if done (e.g., sensitivity or subgroup analyses, meta-regression [see Item 16]). | Not done |
| **DISCUSSION** | | |  |
| Summary of evidence | 24 | Summarize the main findings including the strength of evidence for each main outcome; consider their relevance to key groups (e.g., healthcare providers, users, and policy makers). | Pages 15-17: Definitions describing contacts between livestock and wildlife to be wide-ranging. Making any sort of meaningful comparison between such studies is challenging.  Page 16: Narrow scope of livestock-wildlife contact studies, with the majority of studies focusing on cattle-wildlife contacts and diseases of cattle. Bovine tuberculosis (infection with *M. bovis*) featured prominently, indicative of the economic and potentially zoonotic importance of this disease to the USA and UK, where the most livestock-wildlife contact studies were conducted |
| Limitations | 25 | Discuss limitations at study and outcome level (e.g., risk of bias), and at review-level (e.g., incomplete retrieval of identified research, reporting bias). | Page 18: Our study has some limitations which we summarise here. At present, our generic unified framework does not explicitly account for disease transmission via vectors (e.g. mosquitoes) or fomites (e.g. vehicle tyres), although the latter will to some extent be captured within our definition of indirect contact. We focussed on mammals so did not address diseases such as avian influenza. Small mammals (<5kg) were not included in this review despite bats and some rodents being hosts of pathogens that affect livestock such as Nipah virus, Hendra virus and leptospirosis. While the generic unified framework may be applicable to these types of wildlife, it is unclear whether the observational methods seen in this review would be applicable. |
| Conclusions | 26 | Provide a general interpretation of the results in the context of other evidence, and implications for future research. | Pages 17-19: This review highlights that observing contacts between multiple species is possible and can yield high quality information. Increasing the efficiency of monitoring methods would justify their use for more applications. The generic unified framework presented in this paper is a step towards being able to compare observation methods and contact data in order to standardise and evaluate different monitoring methods. This systematic review of the observational methods used to study contacts, and the subsequent proposal of a generic unified framework for defining contacts, are two steps towards ensuring that data are collected and reported in a standardised way at a time of increasingly urgent need. |
| **FUNDING** | | |  |
| Funding | 27 | Describe sources of funding for the systematic review and other support (e.g., supply of data); role of funders for the systematic review. | SAB is supported by a Bloomsbury PhD studentship and the Royal Veterinary College through the London Interdisciplinary Doctoral Programme (BBSRC project number BB/M009513/1). |

*From:*  Moher D, Liberati A, Tetzlaff J, Altman DG, The PRISMA Group (2009). Preferred Reporting Items for Systematic Reviews and Meta-Analyses: The PRISMA Statement. PLoS Med 6(7): e1000097. doi:10.1371/journal.pmed1000097

For more information, visit: **www.prisma-statement.org**.

Page 2 of 2
