# Supplementary figures and images for: Towards a unified generic framework to define and observe contacts between livestock and wildlife: a systematic review
Source: PeerJ. 2020 Oct 26;8:e10221. doi: 10.7717/peerj.10221 (PMC7594637; doi:10.7717/peerj.10221)

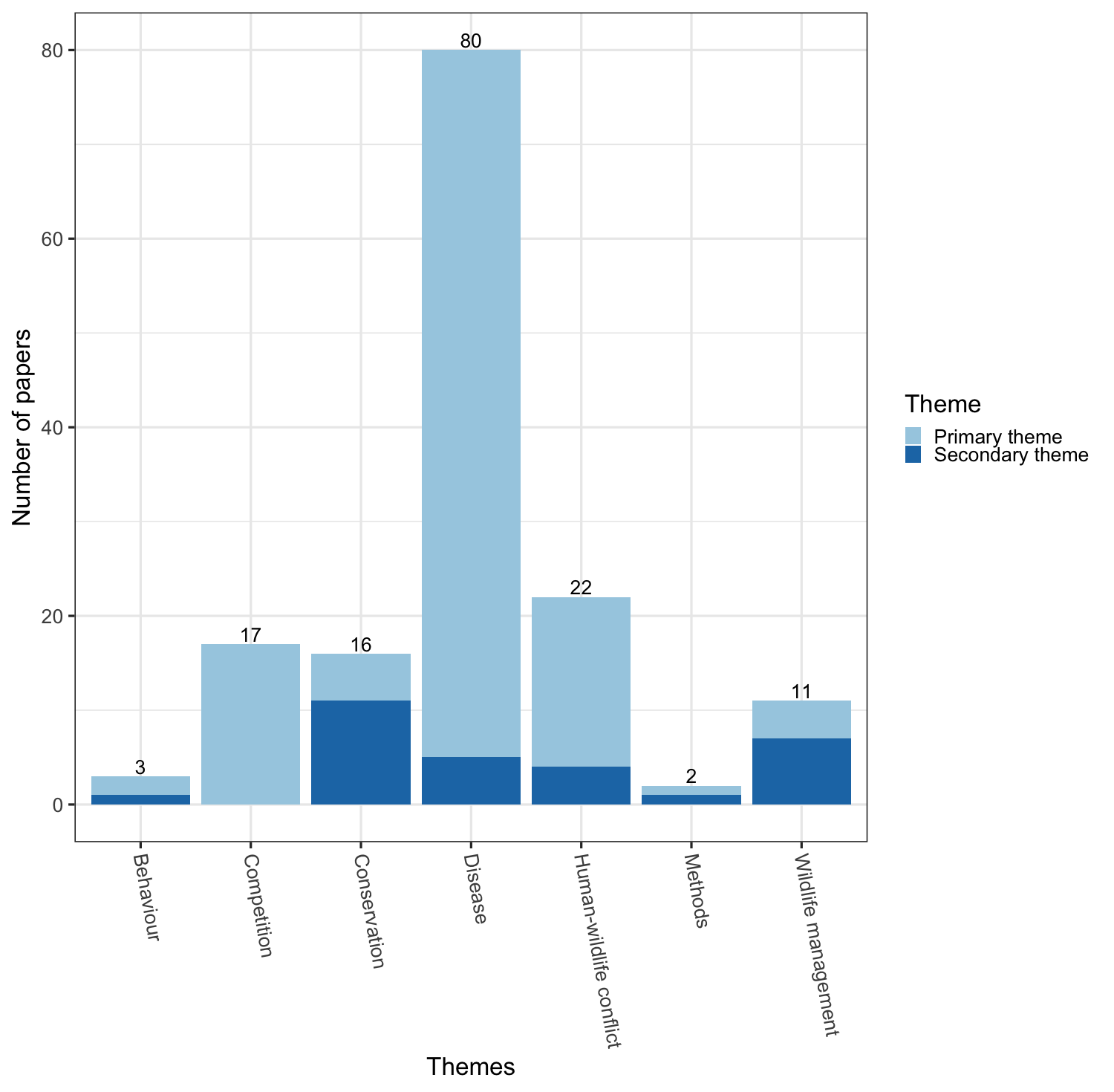

Supplement: Supplemental Information 5 — Livestock-wildlife studies (n = 122) grouped by themes that emerged during data extraction. Where studies had more than one theme, each theme was identified as either primary (main) or secondary (supportive) based on the aims of the study; hence the total number of primary themes in this figure exceeds the number of studies. [file peerj-08-10221-s005.png]

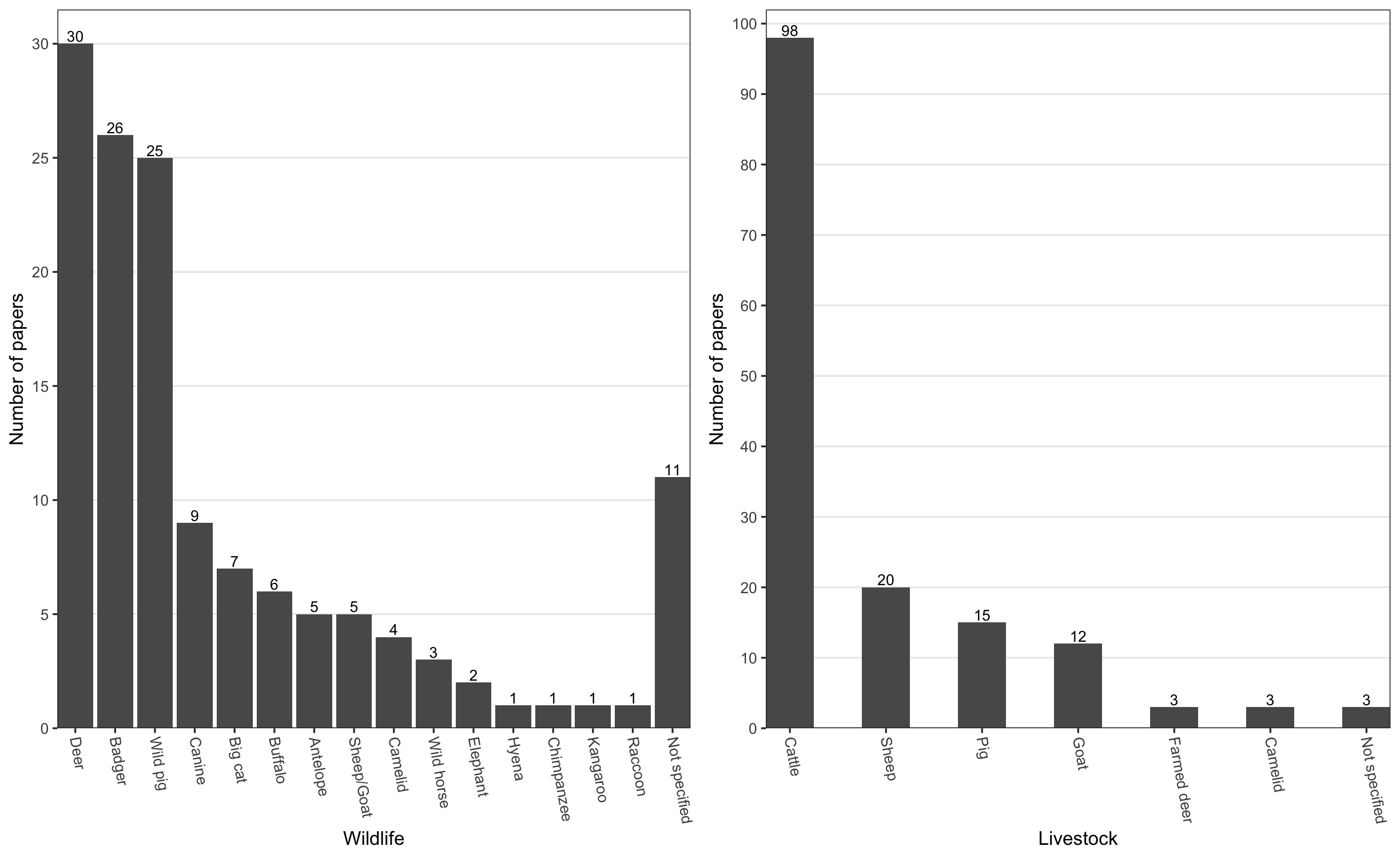

Supplement: Supplemental Information 6 — Many publications monitored multiple species of wildlife and livestock and therefore numbers of studies may exceed 100% for some groups. [file peerj-08-10221-s006.png]

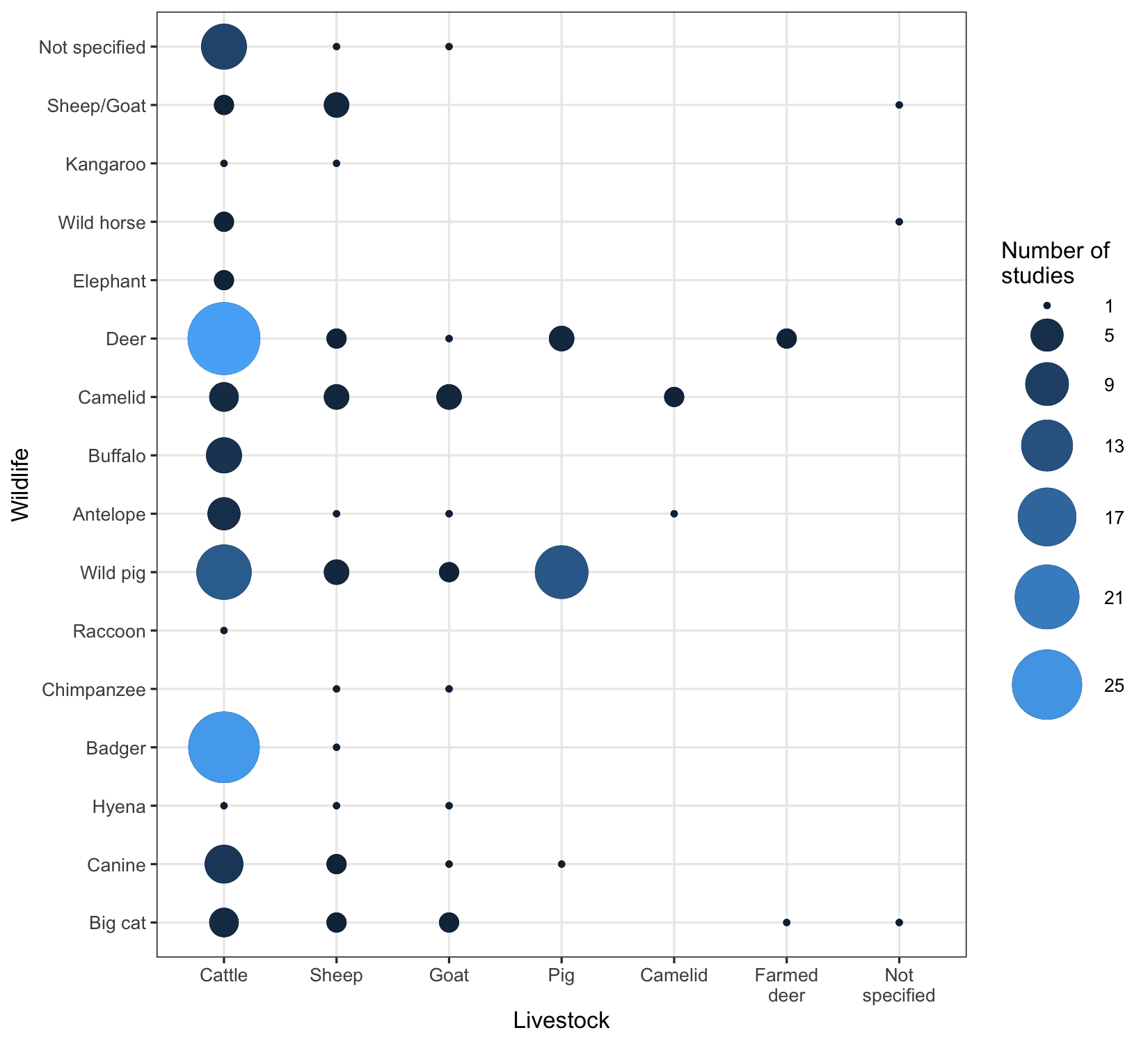

Supplement: Supplemental Information 7 — Data from 122 papers included in the systematic review. The size and shade of circles indicate the number of studies in each category. Many publications used more than one method to monitor contacts, and therefore the numbers of studies exceed 100% for some groups. [file peerj-08-10221-s007.png]

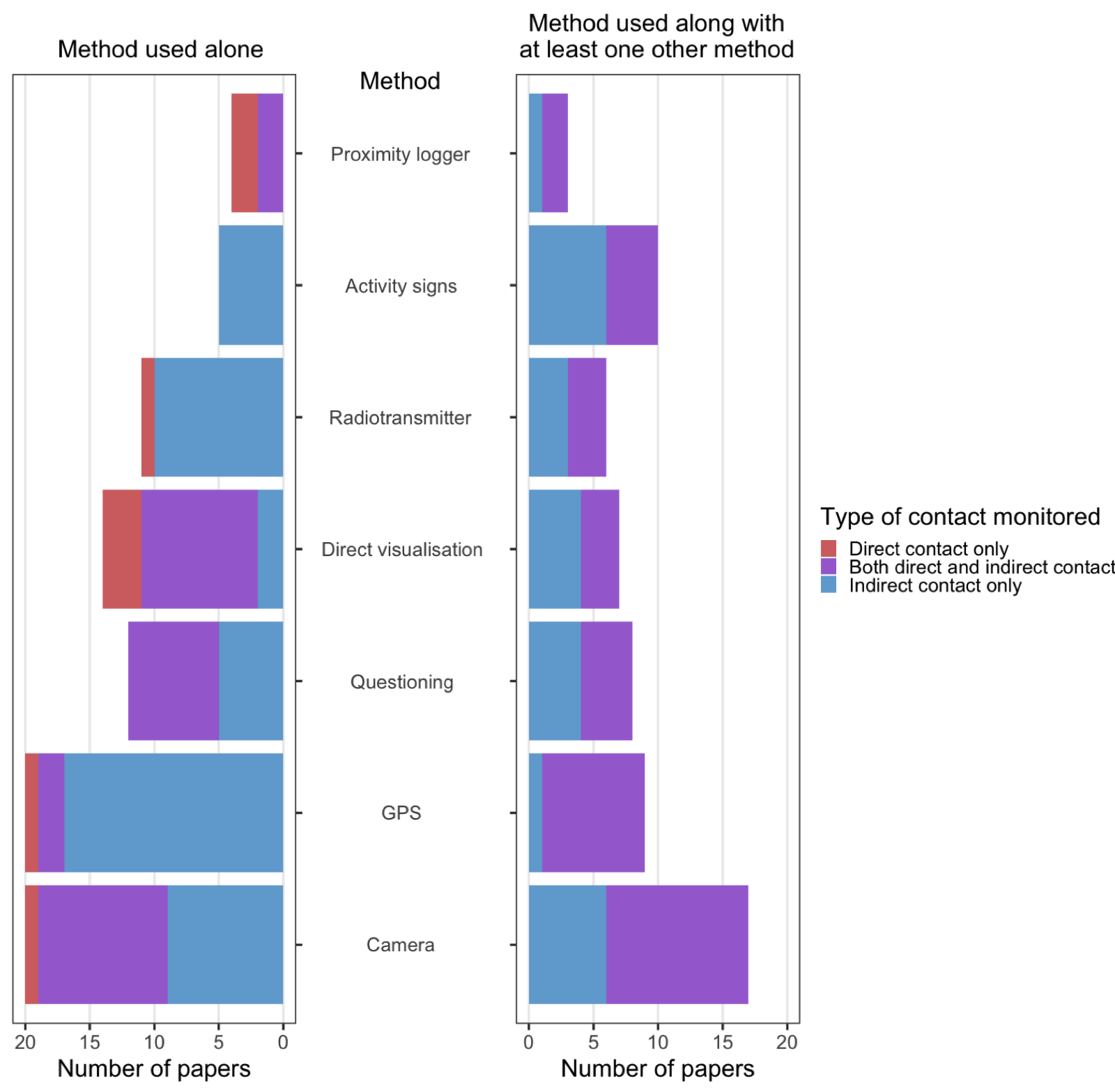

Supplement: Supplemental Information 8 — Methods used to observe wildlife grouped by methods used alone and in combination with other methods, and grouped by whether direct or indirect contact, or both, was monitored. [file peerj-08-10221-s008.png]
