# Supplementary material for: Towards a unified generic framework to define and observe contacts between livestock and wildlife: a systematic review
Source: PeerJ. 2020 Oct 26;8:e10221. doi: 10.7717/peerj.10221 (PMC7594637; doi:10.7717/peerj.10221)
Supplement: Supplemental Information 9 [file peerj-08-10221-s009.docx]

# Pubmed

(wild*[Title/Abstract] OR "free-living"[Title/Abstract] OR "free living"[Title/Abstract] OR "feral"[Title/Abstract] OR "badger"[Title/Abstract] OR "badgers"[Title/Abstract] OR "deer"[Title/Abstract] OR "reindeer"[Title/Abstract] OR "warthog"[Title/Abstract] OR "warthogs"[Title/Abstract] OR "bushpig"[Title/Abstract] OR "bushpigs"[Title/Abstract] OR "bush pig"[Title/Abstract] OR "bush pigs"[Title/Abstract] OR "hog"[Title/Abstract] OR "hogs"[Title/Abstract] OR "antelope"[Title/Abstract] OR "antelopes"[Title/Abstract] OR "buffalo"[Title/Abstract] OR "buffalos"[Title/Abstract] OR "bison"[Title/Abstract] OR "moose"[Title/Abstract] OR "elk"[Title/Abstract] OR "coyote"[Title/Abstract] OR "coyotes"[Title/Abstract] OR "jackal"[Title/Abstract] OR "jackals"[Title/Abstract] OR "carnivore"[Title/Abstract] OR "carnivores"[Title/Abstract] OR "herbivore"[Title/Abstract] OR "herbivores"[Title/Abstract] OR "big cat"[Title/Abstract] OR "big cats"[Title/Abstract] OR "wolf"[Title/Abstract] OR "wolves"[Title/Abstract] OR "dog"[Title/Abstract] OR "dogs"[Title/Abstract] OR "fox"[Title/Abstract] OR "foxes"[Title/Abstract] OR "elephant"[Title/Abstract] OR "elephants"[Title/Abstract] OR "predator"[Title/Abstract] OR "predators"[Title/Abstract] OR "scavenger"[Title/Abstract] OR "scavengers"[Title/Abstract] OR "tiger"[Title/Abstract] OR "tigers"[Title/Abstract] OR "lion"[Title/Abstract] OR "lions"[Title/Abstract] OR "bear"[Title/Abstract] OR "bears"[Title/Abstract] OR "hyena"[Title/Abstract] OR "hyenas"[Title/Abstract] OR "monkey"[Title/Abstract] OR "monkeys"[Title/Abstract] OR "baboons"[Title/Abstract] OR "baboon"[Title/Abstract] OR "leopard"[Title/Abstract] OR "leopards"[Title/Abstract] OR "jaguar"[Title/Abstract] OR "jaguars"[Title/Abstract] OR "rhinoceros"[Title/Abstract] OR "rhinoceroses"[Title/Abstract] OR "rhino"[Title/Abstract] OR "rhinos"[Title/Abstract] OR "hippopotamus"[Title/Abstract] OR "hippopotamuses"[Title/Abstract] OR "hippo"[Title/Abstract] OR "hippos"[Title/Abstract] OR "buffalo"[Title/Abstract] OR "peccary"[Title/Abstract] OR "peccaries"[Title/Abstract] OR “free roaming”[Title/Abstract] OR “free-roaming”[Title/Abstract] OR “bighorn”[Title/Abstract] OR “bighorns”[Title/Abstract] OR “guanaco”[Title/Abstract] OR “guanacos”[Title/Abstract] OR “vicuna”[Title/Abstract] OR “vicunas”[Title/Abstract]) AND (contact*[Title/Abstract] OR visit*[Title/Abstract] OR interact*[Title/Abstract] OR movement*[Title/Abstract] OR "proximity"[Title/Abstract] OR "interface"[Title/Abstract] OR “predation”[Title/Abstract] OR “predations”[Title/Abstract] OR “predate”[Title/Abstract] OR “scavenge”[Title/Abstract] OR "scavenges"[Title/Abstract] OR "scavenging"[Title/Abstract]) AND ("cattle"[Title/Abstract] OR "bovid"[Title/Abstract] OR "bovids"[Title/Abstract] OR "sheep"[Title/Abstract] OR "pig"[Title/Abstract] OR "pigs"[Title/Abstract] OR "swine"[Title/Abstract] OR "suid"[Title/Abstract] OR "suids"[Title/Abstract] OR "goat"[Title/Abstract] OR "goats"[Title/Abstract] OR farm*[Title/Abstract] OR "livestock"[Title/Abstract] OR "ox"[Title/Abstract] OR "oxen"[Title/Abstract] OR "camel"[Title/Abstract] OR "camels"[Title/Abstract] OR "llama"[Title/Abstract] OR "llamas"[Title/Abstract] OR "alpaca"[Title/Abstract] OR "alpacas"[Title/Abstract] OR "domestic"[Title/Abstract])

(“wildlife-livestock”[Title/Abstract]) AND (contact*[Title/Abstract] OR visit*[Title/Abstract] OR interact*[Title/Abstract] OR movement*[Title/Abstract] OR "proximity"[Title/Abstract] OR "interface"[Title/Abstract] OR “predation”[Title/Abstract] OR “predations”[Title/Abstract] OR “predate”[Title/Abstract] OR “scavenge”[Title/Abstract] OR "scavenges"[Title/Abstract] OR "scavenging"[Title/Abstract])

# Scopus

TITLE-ABS (wild* OR "free-living" OR "free living" OR "feral" OR "badger" OR "badgers" OR "deer" OR "reindeer" OR "warthog" OR "warthogs" OR "bushpig" OR "bushpigs" OR "bush pig" OR "bush pigs" OR "hog" OR "hogs" OR "antelope" OR "antelopes" OR "buffalo" OR "buffalos" OR "bison" OR "moose" OR "elk" OR "coyote" OR "coyotes" OR "jackal" OR "jackals" OR "carnivore" OR "carnivores" OR "herbivore" OR "herbivores" OR "big cat" OR "big cats" OR "wolf" OR "wolves" OR "dog" OR "dogs" OR “fox” OR “foxes” OR "elephant" OR "elephants" OR "predator" OR "predators" OR "scavenger" OR "scavengers" OR "tiger" OR "tigers" OR "lion" OR "lions" OR "bear" OR "bears" OR "hyena" OR "hyenas" OR "monkey" OR "monkeys" OR "baboons" OR "baboon" OR "leopard" OR "leopards" OR "jaguar" OR "jaguars" OR "rhinoceros" OR "rhinoceroses" OR "rhino" OR "rhinos" OR "hippopotamus" OR "hippopotamuses" OR "hippo" OR "hippos" OR "buffalo" OR "peccary" OR "peccaries" OR "free-roaming" OR "free roaming" OR "bighorn" OR “bighorns” OR “guanaco” OR “guanacos” OR “vicuna” OR “vicunas”) AND TITLE-ABS (contact* OR visit* OR interact* OR movement* OR "proximity" OR "interface” OR “predation” OR “predations” OR “predate” OR “scavenge” OR "scavenges" OR "scavenging") AND TITLE-ABS ("cattle" OR "bovid" OR "bovids" OR "sheep" OR "pig" OR "pigs" OR "swine" OR "suid" OR "suids" OR "goat" OR "goats" OR farm* OR "livestock" OR "ox" OR "oxen" OR "camel" OR "camels" OR "llama" OR "llamas" OR "alpaca" OR "alpacas" OR "domestic")

TITLE-ABS ("wildlife-livestock") AND TITLE-ABS (contact* OR visit* OR interact* OR movement* OR "proximity" OR "interface" OR "predation" OR "predations" OR "predate" OR "scavenge" OR "scavenges" OR "scavenging")

# CAB Abstracts

(title:(wild* OR "free-living" OR "free living" OR "feral" OR "badger" OR "badgers" OR "deer" OR "reindeer" OR "warthog" OR "warthogs" OR "bushpig" OR "bushpigs" OR "bush pig" OR "bush pigs" OR "hog" OR "hogs" OR "antelope" OR "antelopes" OR "buffalo" OR "buffalos" OR "bison" OR "moose" OR "elk" OR "coyote" OR "coyotes" OR "jackal" OR "jackals" OR "carnivore" OR "carnivores" OR "herbivore" OR "herbivores" "big cat" OR "big cats" OR "wolf" OR "wolves" OR "dog" OR "dogs" OR “fox” OR “foxes” OR "elephant" OR "elephants" OR "predator" OR "predators" OR "scavenger" OR "scavengers" OR "tiger" OR "tigers" OR "lion" OR "lions" OR "bear" OR "bears" OR "hyena" OR "hyenas" OR "monkey" OR "monkeys" OR "baboons" OR "baboon" OR "leopard" OR "leopards" OR "jaguar" OR "jaguars" OR "rhinoceros" OR "rhinoceroses" OR "rhino" OR "rhinos" OR "hippopotamus" OR "hippopotamuses" OR "hippo" OR "hippos" OR "buffalo" OR "peccary" OR "peccaries" OR "free-roaming" OR "free roaming" OR "bighorn" OR "bighorns" OR "guanaco" OR "guanacos" OR “vicuna” OR “vicunas”) OR ab:(wild* OR "free-living" OR "free living" OR "feral" OR "badger" OR "badgers" OR "deer" OR "reindeer" OR "warthog" OR "warthogs" OR "bushpig" OR "bushpigs" OR "bush pig" OR "bush pigs" OR "hog" OR "hogs" OR "antelope" OR "antelopes" OR "buffalo" OR "buffalos" OR "bison" OR "moose" OR "elk" OR "coyote" OR "coyotes" OR "jackal" OR "jackals" OR "carnivore" OR "carnivores" OR "herbivore" OR "herbivores" OR "big cat" OR "big cats" OR "wolf" OR "wolves" OR "dog" OR "dogs" OR “fox” OR “foxes” OR "elephant" OR "elephants" OR "predator" OR "predators" OR "scavenger" OR "scavengers" OR "tiger" OR "tigers" OR "lion" OR "lions" OR "bear" OR "bears" OR "hyena" OR "hyenas" OR "monkey" OR "monkeys" OR "baboons" OR "baboon" OR "leopard" OR "leopards" OR "jaguar" OR "jaguars" OR "rhinoceros" OR "rhinoceroses" OR "rhino" OR "rhinos" OR "hippopotamus" OR "hippopotamuses" OR "hippo" OR "hippos" OR "buffalo" OR "peccary" OR "peccaries" OR "free-roaming" OR "free roaming" OR "bighorn" OR "bighorns" OR "guanaco" OR "guanacos" OR “vicuna” OR “vicunas”)) AND (title:(contact* OR visit* OR interact* OR movement* OR "proximity" OR "interface" OR “predation” OR “predations” OR “predate” OR “scavenge” OR "scavenges" OR "scavenging") OR ab:(contact* OR visit* OR interact* OR movement* OR "proximity" OR "interface" OR “predation” OR “predations” OR “predate” OR “scavenge” OR "scavenges" OR "scavenging")) AND (title:("cattle" OR "bovid" OR "bovids" OR "sheep" OR "pig" OR "pigs" OR "swine" OR "suid" OR "suids" OR "goat" OR "goats" OR farm* OR "livestock" OR "ox" OR "oxen" OR "camel" OR "camels" OR "llama" OR "llamas" OR "alpaca" OR "alpacas" OR "domestic") OR ab:("cattle" OR "bovid" OR "bovids" OR "sheep" OR "pig" OR "pigs" OR "swine" OR "suid" OR "suids" OR "goat" OR "goats" OR farm* OR "livestock" OR "ox" OR "oxen" OR "camel" OR "camels" OR "llama" OR "llamas" OR "alpaca" OR "alpacas" OR "domestic"))

(title:("wildlife-livestock") OR ab:("wildlife-livestock")) AND (title:(contact* OR visit* OR interact* OR movement* OR "proximity" OR "interface" OR "predation" OR "predations" OR "predate" OR "scavenge" OR "scavenges" OR "scavenging") OR ab:(contact* OR visit* OR interact* OR movement* OR "proximity" OR "interface" OR "predation" OR "predations" OR "predate" OR "scavenge" OR "scavenges" OR "scavenging"))
