# Supplementary material for: Towards a unified generic framework to define and observe contacts between livestock and wildlife: a systematic review
Source: PeerJ. 2020 Oct 26;8:e10221. doi: 10.7717/peerj.10221 (PMC7594637; doi:10.7717/peerj.10221)
Supplement: Supplemental Information 10 [file peerj-08-10221-s010.docx]

Attali, D. and C. Baker, ggExtra: Add Marginal Histograms to 'ggplot2', and More 'ggplot2' Enhancements. R package version 0.9., 2019. https://CRAN.R-project.org/package=ggExtra

Baptiste, A., gridExtra: Miscellaneous Functions for "Grid" Graphics. R package version 2.3., 2017. https://CRAN.R-project.org/package=gridExtra

Comtois, D., summarytools: Tools to Quickly and Neatly Summarize Data. R package version 0.9.6, 2020. https://CRAN.R-project.org/package=summarytools

Hadley W., et al., dplyr: A Grammar of Data Manipulation. R package version 0.8.5., 2020 https://CRAN.R-project.org/package=dplyr

Hadley, W., ggplot2: Elegant Graphics for Data Analysis. Springer-Verlag New York, 2016. ISBN 978-3-319-24277-4, https://ggplot2.tidyverse.org.

Hadley W., stringr: Simple, Consistent Wrappers for Common String Operations. R package version 1.4.0., 2019. https://CRAN.R-project.org/package=string

Hadley, W. and L. Henry, tidyr: Tidy Messy Data. R package version 1.0.2., 2020.

https://CRAN.R-project.org/package=tidyr

Kassambara, A., ggpubr: 'ggplot2' Based Publication Ready Plots. R package version 0.2.5., 2020. https://CRAN.R-project.org/package=ggpubr
