# Supplementary material for: Towards a unified generic framework to define and observe contacts between livestock and wildlife: a systematic review
Source: PeerJ. 2020 Oct 26;8:e10221. doi: 10.7717/peerj.10221 (PMC7594637; doi:10.7717/peerj.10221)
Supplement: Supplemental Information 11 [file peerj-08-10221-s011.docx]

| Continent | Publications (%) | % Cumulative |
| --- | --- | --- |
| Europe | 49 (40) | 40 |
| North America | 33 (27) | 67 |
| Africa | 27 (22) | 89 |
| South America | 6 (5) | 94 |
| Asia | 4 (3) | 97 |
| Australasia | 3 (2) | 100 |
| Total | 122 (100) |  |
