# Supplementary material for: Towards a unified generic framework to define and observe contacts between livestock and wildlife: a systematic review
Source: PeerJ. 2020 Oct 26;8:e10221. doi: 10.7717/peerj.10221 (PMC7594637; doi:10.7717/peerj.10221)
Supplement: Supplemental Information 12 [file peerj-08-10221-s012.docx]

| Bacterial Disease | Publications (%) | % Cumulative |
| --- | --- | --- |
| Bovine tuberculosis | 49 (84) | 84 |
| Brucellosis | 4 (7) | 91 |
| Pasteurellosis | 2 (3) | 95 |
| Mycoplasmosis | 1 (2) | 97 |
| Non-specific | 2 (3) | 100 |
| Total | 58 (100) |  |

Data from the 122 papers included in the systematic review.
