# Supplementary material for: Towards a unified generic framework to define and observe contacts between livestock and wildlife: a systematic review
Source: PeerJ. 2020 Oct 26;8:e10221. doi: 10.7717/peerj.10221 (PMC7594637; doi:10.7717/peerj.10221)
Supplement: Supplemental Information 13 [file peerj-08-10221-s013.docx]

| Viral Diseases | Publications (%) | % Cumulative |
| --- | --- | --- |
| Foot and mouth disease | 8 (62) | 612 |
| African swine fever | 2 (15) | 77 |
| Pseudorabies | 2 (15) | 92 |
| Malignant catarrhal fever | 1 (7) | 100 |
| Total | 13 (100) |  |

Data from the 122 papers included in the systematic review.
